# The effect of membrane exposure on lateral ridge augmentation: a case-controlled study

**DOI:** 10.1186/s40729-017-0089-z

**Published:** 2017-06-22

**Authors:** Mehmet A. Eskan, Marie-Eve Girouard, Dean Morton, Henry Greenwell

**Affiliations:** 1 Sisli, Istanbul, Turkey; 2 Sherbrooke, Québec Canada; 3 Department of Prosthodontics, Indiana University School of Dentistry, Indianapolis, IN 46202 USA; 4 Department of Oral Health and Rehabilitation, Division of Periodontics, University of Louisville School of Dentistry, Louisville, KY 40292 USA; 5 Clinic Eska, Terrace Fulya, Tesvikiye Mah., Hakki Yeten Cad, Sisli, Istanbul, Turkey

**Keywords:** Graft loss, Lateral ridge augmentation, Matrix barrier, Membrane exposure

## Abstract

**Background:**

The effect of membrane exposure on guided bone regeneration (GBR) for lateral ridge augmentation has been poorly addressed. This case-controlled study aimed to investigate potential effect of membrane exposure lateral ridge augmentation and subsequent implant placement.

**Methods:**

A total of 14 patients that did receive lateral ridge augmentation procedure using allogeneic cancellous graft particulate in combination with an alloplastic bioresorbable matrix barrier were retrospectively selected for this study. Bone width was measured at the crest with a digital caliper before bone augmentation and at the reopening for implant placement 4 months later for all patients. Cases where primary flap closure was achieved and the barrier did not expose throughout the time until implant placement were assigned to the control group (*n* = 7). Cases where primary closure could not be achieved or a barrier exposure happened within the first week following the initial surgery were assigned to the test group.

**Results:**

The measured alveolar ridge width before surgery as well as after GBR procedure were not statistically significant different between the two groups (*p* > 0.05). Both groups showed a significant (*p* < 0.05) increase in their mean alveolar ridge width 4 months after later augmentation procedure, from 3.4 ± 1.2 to 6.0 ± 1.1 mm in the control group and from 3.6 ± 1.0 to 5.0 ± 1.4 mm in the test group. However, the mean alveolar ridge gain was significantly greater in the control group than in the test group (*p* < 0.05). Consequently, the reduction of the augmented alveolar ridge was significantly higher in the test group averaging to 4.7 mm than for the control group showing a loss of 3.1 mm after 4 months, respectively. However, in all 14 cases, successful implant placement was achieved after 4 months.

**Conclusions:**

Within the limit of this study, it can be concluded that early exposure of a bioresorbable matrix barrier during lateral ridge augmentation may compromise the results of the GBR procedure but may still result in a favorable alveolar ridge width gain that allows for the placement of dental implants.

## Background

It has been reported that unpreserved alveolar ridges can show substantial horizontal and/or vertical ridge deficiency [1, 2] that lack the sufficient alveolar ridge dimensions to allow the ideal positioning of the implant and enhance long-term prognosis of the clinical outcomes [3]. Guided bone regeneration (GBR) is a predictable technique for augmenting the alveolar ridge width that has been used for more than two decades, and osseointegration and long-term implant survival rate have been reported to be similar in grafted areas than in native bone [4, 5].

One of the main components in GBR procedures is the use of a resorbable or non-resorbable barrier membranes that stabilize the bone grafting material and protect it from the ingrowth of surrounding soft tissues [6, 7]. Therefore, non-resorbable PTFE membranes have been developed for GBR that present an inner occlusive surface to prevent migration of epithelial and fibroblast cells into the defect and to maintain adequate space for bone formation and wound stabilization [8]. However, PTFE membrane might lead to compromised vascular supply of the flaps [9] and exhibited a higher incidence of premature membrane exposures [8, 10, 11] as well as gingival recession [12], which might cause an esthetic problems in the anterior regions.

It is well know that primary closure is increasing the clinical outcome of the GBR procedures [6]. To overcome membrane exposure, it has been suggested to perform a periosteal releasing incision [13]. However, periosteal releasing incisions might cause more swelling, bleeding, and patient discomfort. Importantly, they also may compromise blood circulation [14], and re-positioning flap coronally can result in a misaligned mucogingival line (MGL) if not properly performed [13]. This misaligned MGL might also cause esthetic problems especially in the anterior regions. Therefore, the use of resorbable membrane in the patients might be beneficial, especially in patients with thin soft tissue biotypes.

Various resorbable membranes exist in the market composing of dura mater, poly-lactic acid, polyglycolic acid, polyurethane, or mostly collagen. Still, even resorbable membranes show frequent events of membrane exposures after GBR procedures. For example, between 22 and 32% of early membrane exposure have been reported for collagen membrane by several authors [15–18]. A major drawback of collagen membrane might be that lose their integrity in 1 week [18] when exposed to the proteolytic environment of the oral cavity that leaves the graft material unprotected and can lead to graft loss.

Alloplastic barriers have been proposed as dental membranes for regenerative dentistry that show slower degradation but still good biocompatibility [19–21]. Among those, bioresorbable matrix barrier has been developed for periodontal regeneration and showed effectiveness to reduce epithelial down-growth while promoting the formation of periodontal ligament and alveolar bone in various clinical studies [19, 22–25]. However, the documentation of the performance of bioesorbable matrix barrier in GBR procedures is spares [26–29] and their performance in the case of matrix exposure remains elusive.

Therefore, this case-controlled study aims to investigate the effectiveness of GUIDOR *bioresorbable matrix barrier* for lateral bone augmentation procedures and the effect of exposures on its performance.

## Methods

Fourteen subjects were retrospectively recruited for this case-controlled study. In test group (seven patients), primary closure was not achieved and membrane was left exposed at the initial surgery or it became exposed during the first week of healing. In the control group (seven patients), primary wound closure was achieved and no exposure of the membrane occurred until the placement of a dental implant 4 months after augmentation. Each patient received a particulate cancellous allograft (500 to 800 μm, RegenerOss, BioMet 3i), and then, the grafted defect area was covered with a bioresorbable matrix membrane (Sunstar, Suisse SA, Etoy, Switzerland). Longer span edentulous spaces were divided into individual sites based on a 10–12-mm width per site, and each site was bordered by at least one tooth. The subject inclusion criteria included a treatment that was planned to receive a dental implant in the future. At least 18-year-old males and females were included in this study. All subjects signed an informed consent approved by the University of Louisville Institutional Review Board in July 2010. Exclusion criteria excluded patients with uncontrolled diabetes, who are smokers, and with immune diseases or other systemic diseases that significantly affect the periodontium; patients with an allergy to any material or medication used in this study; and patients who need prophylactic antibiotics, previous head and neck radiation therapy, and chemotherapy in the previous 12 months and with severe psychological problems.

### Surgical treatment

**Fig. 1:**
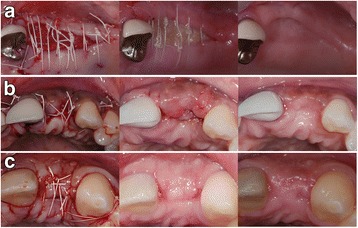
Clinical photographs of the both treatment groups after the initial surgery, 1 week post-op and at the re-entry. **a**) In the test group, no primary wound closure was achieved (*left*) and the barrier was left exposed for secondary intention healing. After 1 week, the matrix remained exposed (*middle*) showing no signs of infection. For months later, the exposed area was covered by a keratinized tissue (*right*). **b**) In the test group, primary wound closure was achieved at surgery (*left*). However, the barrier became exposed after 1 week of healing (*middle*). For months later, exposed area was covered with a keratinized tissue (*right*). **c**) In the control group, primary wound closure was achieved (*left*). After 1 week (*middle*), primary healing happened without any signs of membrane exposure. For months later, the site healed uneventfully (*right*)

All surgical procedures were done by one surgeon (ME). The surgical procedure consisted of the reflection of a full thickness flap to expose the residual alveolar ridge. Following complete exposure of the defect area, horizontal ridge width was measured with a digital caliper at the midridge crestal level. Horizontal ridge measurements (at the crestal level) included initial ridge, initial augmented ridge (residual ridge plus graft), and the ridge at the re-entry after the 4-month healing time. All measurements were performed by a masked examiner (MEG), who was unaware of the treatments. Cortical perforations were performed with a ½ round bur to increase vascularization in the defect area [30]. The bone screws (Salvin Dental Specialties, Charlotte, NC) and cancellous particulate allograft were placed to allow for augmentation to achieve 8 mm horizontal width available for implant placement. Horizontal measurements were again taken with the same caliper. Then, the grafted area was covered with a bioresorbable matrix barrier. In the buccal surface, the graft material was completely covered by the membrane. However, in the lingual or palatal surface, the membrane was tucked least 5 mm between the alveolar bone and gingival tissue. Periosteal releasing incisions were performed when needed, and all wound was tried to close primarily and flap re-positioned coronally without compromising MGL. In the test group, the membrane could not be closed primarily (Fig. 1a) or became exposed to the oral environment in 1 week after primary closure was achieved during surgery (Fig. 1b). In the control group, primary closure was achieved with a monofilament suture (Cytoplast, PTFE Suture, Osteogenics Biomedical, Lubbock, TX) (Fig. 1c). The patients were prescribed doxycycline hyclate 50 mg once a day for 2 weeks and hydrocodone when needed. Sutures were removed in 10 days. The subjects were seen every other week to clean the area with hydrogen peroxide.

### Statistical analysis

Means ± SD was calculated for all parameters. The statistical significance difference of means between the groups was tested using an exact two-sample Fisher-Pitman permutation test; since the sample size seemed too small to test for normality, *p* < 0.05 was considered to be significant.

## Results

### The effect of early membrane exposure on alveolar ridge width changes

To assess if the baseline situations of the patients in the two treatment groups were comparable and well balanced, the distribution of gender, age, and the initial ridge measurements were compared. There were three women and four men in each group. The median age for the test and control group was 50 and 62 years old, respectively (Table 1). The initial alveolar mean ridge widths before lateral augmentation in the test and control group were 3.6 ± 1.0 and 3.4 ± 1.2 mm, respectively (Table 2). Therefore, the baseline situation of the two groups was comparable (*p* > 0.05).

**Table 1:** Patient population and demographics and sites

Groups	Subject no.	Sex	Site	Age
Exposed (test) group	1	Female	13	74
	2	Male	6	62
	3	Female	29	62
	4	Male	12	62
	5	Male	8	59
	6	Male	19	29
	7	Female	9	23
Non-exposed (control) group	8	Female	11	60
	9	Female	30	68
	10	Male	30	68
	11	Male	9	50
	12	Male	25	42
	13	Female	10	39
	14	Male	9	25

In the control (non-membrane exposure) and test group (membrane exposure), the subject’s age, sex, and defect areas are presented

**Table 2:** Baseline and re-entry measurement of the alveolar ridge width

Groups	Initial ridge width (mm)	Ridge width at re-entry (mm)	Ridge width gain (mm)
Exposed (tests)	3.6 ± 1.0	5.0 ± 1.4	1.4 ± 1.0
Non-exposed (control)	3.4 ± 1.2	6.0 ± 1.1	2.6 ± 1.0
Fisher-Pitman permutation	*p =* 1.00	*p =* 0.168	*p =* 0.047

At the entry and re-entry, the alveolar ridge width was measured using a digital caliper at the crestal level in both groups. In the control group (non-exposure), the mean ridge width was 3.4 mm and changed to 6.0 mm (*p* < 0.01). In the test group, the mean of ridge was 3.6 mm and changed to 5.0 mm. *p* values that were calculated for between mean groups analysis are displayed. Alveolar ridge gain was calculated by subtracting re-entry measurement from the entry measurement for each patient at the crestal level using a digital caliper. In the control group, the mean of the gain was 2.6 mm, while it was 1.4 mm in the test group. *p* values for between-groups analysis are displayed

No infection, discomfort, or suppuration was reported for neither of the two groups throughout the study, and all surgical sites did heal uneventfully. The initial mean ridge width before lateral augmentation of the control group increased from 3.4 ± 1.2 to 6.0 ± 1.1 mm at the 4-month re-entry (Table 2). The initial mean ridge width before lateral augmentation increased from 3.6 ± 1.0 to 5.0 ± 1.4 mm at the 4-month re-entry in the test group (Table 2). This led to an alveolar mean ridge gain of 1.4 ± 1.0 mm in the test group and 2.6 ± 1.0 mm in the control group. Both groups did show a statistically significant (*p* < 0.05) ridge width gain between baseline and at the 4-month re-entry (Table 2). However, the results showed that early exposure (test group) resulted in significant (*p* < 0.05) less gain of the alveolar ridge width than when the membrane was not exposed (Table 2).

### The effect of early membrane exposure on graft reduction

Furthermore, the reduction of the augmented ridge width right after the lateral augmentation (baseline) to implant placement (after 4 months) was assessed in each subjects. The mean ridge width after lateral ridge augmentation procedure was 9.7 ± 0.9 mm for the test group and 9.1 ± 0.8 mm for the control group (Table 3). The difference between the groups were not statically significant (Table 3). Therefore, baseline situations of the two groups were comparable. Regardless the membrane exposure, there was a significant (*p* > 0.05) reduction of the initial later ridge augmentation in the both groups after the 4-month healing time. However, the augmented ridge width reduction of 4.7 ± 1.4 mm in the test group was significantly higher (*p* < 0.05) than the 3.1 ± 0.9 mm assessed for the control group (Table 3). The percentage of ridge width reduction was 48 ± 13% in the test group compared to 33 ± 10% in the control group. Therefore, early membrane exposure resulted in higher reduction of the augmented ridge.

**Table 3:** Alveolar ridge width reduction

Groups	Grafted ridge width	Ridge width at the re-entry	Grafted ridge reduction (mm)
Exposed (test)	9.7 ± 0.9	5.0 ± 1.4	4.7 ± 1.4
Non-exposed (control)	9.1 ± 0.8	6.0 ± 1.1	3.1 ± 0.9
Fisher-Pitman	*p* = 1.00	*p =* 0.260	*p =* 0.030

The residual alveolar ridge width plus graft width was measured at the crestal level at the entry and re-entry procedure. In the test and control group, the mean of the grafted width was 9.7 and 9.1 mm, respectively. Graft reduction was 4.7 and 3.1 mm for the test and control group, respectively. The percentage of the graft reduction was calculated using the formula: ([Amount of graft reduction/Grafted alveolar bone width] × 100. *p* values for between-groups analysis are displayed

## Discussion

Although numerous studies in the literature show successful outcomes of the GBR procedure [6, 31], the most common clinical complication in GBR procedures is early membrane exposure [9]. There is a general clinical impression that the ridge augmentation results are compromised in the case of early membrane exposures [32, 33]. In this case-controlled study, which was based on a patient subset from our previous randomized clinical trial, the clinical effect of exposure of a bioresorbable matrix membrane was evaluated [27]. Based on clinical ridge width dimension measurements, a mean ridge width gain of 1.4 and 2.6 mm were calculated for the test and the control group, respectively. On the other hand, a reduction of 4.7 and 3.1 mm of the initially augmented ridge width was measured for the test and control group, respectively. Together, these results clearly indicated that the early membrane exposure in lateral ridge augmentation procedure resulted in significantly lower ridge width gain probably due to a significant higher resorption of the augmented graft during the healing process.

Still, the ridge width gain in both groups was sufficient to allow for the successful placement of dental implants in all 14 subjects without any complication. The exposed matrix barrier degraded within 6–7 weeks or was covered by soft tissue without any further complications. This observed degradation time is markedly longer than that of collagen membrane, which is reported to be completely resorbed 1 to 2 weeks after exposure [18, 34]. The prolonged degradation time of matrix barrier seems to provide prolonged protecting of the underlying graft supporting the bone regeneration process. During this healing process, all exposures did resolve within 6–7 weeks and no membrane had to be extracted. During this period, the exposed bioresorbable matrix barrier became covered with keratinized tissue over time. The secondary healing in exposed area lead to a subsequent increase in the width of keratinized tissue superior to the band of keratinized tissue observed in the control group (Fig. 1a). This shows the epithelization nor the subsequent keratinization process was not altered by an inflammatory situation that could have been triggered by the presence of the matrix barrier or its degradation product. This demonstrated the good healing properties of this barrier membrane. However, the gain of keratinized tissue was not quantitatively measured; thus, this is a clinical observation rather than a documented outcome. The predictability of gaining both keratinized tissue and horizontal ridge dimension simultaneously needs further investigation to confirm this observation. The other main advantage using a bioresorbable matrix barrier over non-resorbable PTFE membrane in the GBR procedures was that all exposures did resolve within 6–7 weeks without any complications and without the need of second surgery to extract the barrier. This might be an important advantage in the patient showing a thin biotype and in situations where primary closure is difficult to achieve in the GBR procedures.

The microbial contamination of the matrix barrier during exposure could be another important factor that might hamper bone formation within the underlying graft. This factor has not been investigated in the present study. However, it has been reported by other groups that the resorbable matrix barrier per se might be less prone to bacterial contamination and can be better cleaned using disinfectant agents such as chlorhexidine rinse than PTFE membranes [35]. Matrix membrane presents an outer and inner surface. The external surface is more occlusive (the pore sizes are bigger than those of internal surface) to allow gingival tissue penetration. The internal layer, smaller pores, prevents further penetration of the gingival tissue through the barrier, thus protecting new bone formation underneath the barrier. From clinical observation, the space between the two layers seemed already occupied by connective tissue protecting the inner layer and leaving only the outer layer of the matrix exposed to the oral cavity and subsequent degradation. Still, this clinical observation has to be confirmed in further studies.

The results from this study suggest that primary flap closure over the matrix barrier is preferable leading to better ridge width gain than when the matrix is left exposed or early exposures happen. However, exposures were not completely detrimental to the lateral ridge augmentation and sufficient ridge width gain could be achieved allowing for successful implant placement. In critical cases, where 1 or 2 mm less bone would affect the esthetic results, the matrix barrier should not be left exposed and due care should be taken to avoid any exposures during healing after primary closure was achieved.

## Conclusions

Within the limits of this case-controlled study, it can be concluded that lateral ridge augmentation procedures in atrophic alveolar ridges using bioresorbable matrix barriers without achieving primary flap closure or in the case of early exposures can still lead to clinically satisfying ridge width gain that allows for the placement of dental implants. However, exposures seem to limit the ridge width gain. Therefore, in esthetic challenging situations, efforts should be made to achieve primary wound closure and to avoid subsequent membrane exposure.
